# Supplementary material for: The Pitfalls of Heterosis Coefficients
Source: Plants (Basel). 2020 Jul 9;9(7):875. doi: 10.3390/plants9070875 (PMC7412094; doi:10.3390/plants9070875)
Supplement: Supplementary file 1 [file plants-09-00875-s001.zip › plants-835059_Suppl_Materials/plants-835059-english_Supp_data.pdf]

plants

MDPI

Commentary

The Pitfalls of Heterosis Coefficients: Supplementary Materials

Dominique de Vienne * and Julie B. Fiévet

GQE—Le Moulon, INRAE, Université Paris-Saclay, CNRS, AgroParisTech, 91190 Gif-sur-Yvette, France; julie.fievet@inrae.fr

* Correspondence: dominique.de-vienne@inrae.fr; Tel.: +33-1-69332360

Received: 29 May 2020; Accepted: 4 July 2020; Published: date

check for updates

Table S1

Heterosis coefficients expressed as functions of genetic effects. Subscripts: same as in Table 1. μ, mean of the multilocus homozygous genotypes; $\sum a$, sum of the additive effects; $\sum d$, sum of the dominance effects; $\sum e_{\text{dom}}$, sum of the dominance-by-dominance epistatic effects; $\sum e_{\text{add}}$, sum of the additive-by-additive epistatic effects; $\sum e_{\text{add even}}$, sum of the additive-by-additive epistatic effects involving an even number of genes; $\sum e_{\text{add odd}}$, sum of the additive-by-additive epistatic effects involving an odd number of genes (from [13]).

Reference	Coefficient	Coefficient as function of genetic effects
Mid-parent	$H_{\text{mp}} = z_{12} - \bar{z}$	$\sum d + \sum e_{\text{dom}} - \sum e_{\text{add even}}$
	$H_{\text{MP}} = \dfrac{z_{12} - \bar{z}}{\bar{z}}$	$\dfrac{\sum d + \sum e_{\text{dom}} - \sum e_{\text{add even}}}{\mu + \sum e_{\text{add even}}}$
	$H_{\text{PR}} = \dfrac{z_{12} - \bar{z}}{(z_2 - z_1)/2}$	$\dfrac{\sum d + \sum e_{\text{dom}} - \sum e_{\text{add even}}}{\sum a + \sum e_{\text{add odd}}}$
Best-parent	$H_{\text{bp}} = z_{12} - z_2$	$\sum d + \sum e_{\text{dom}} - \sum a - \sum e_{\text{add}}$
	$H_{\text{BP}} = \dfrac{z_{12} - z_2}{z_2}$	$\dfrac{\sum d + \sum e_{\text{dom}} - \sum a - \sum e_{\text{add}}}{\mu + \sum a + \sum e_{\text{add}}}$

Figure S1

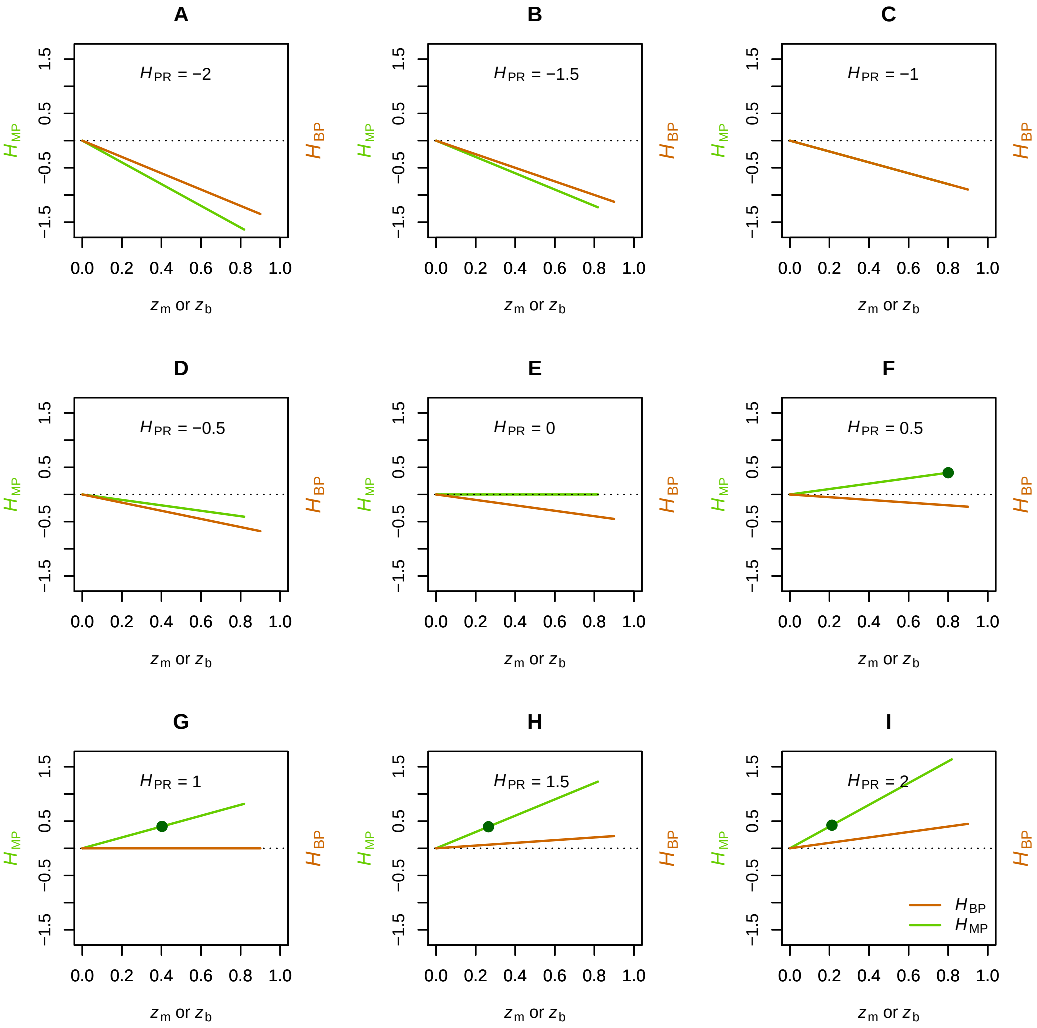

Influence of the scale of the parental values on H_{MP} and H_{BP} for different values of the potence ratio H_{PR}. (A) to (I) H_{PR} values from −2 to 2. $z_m = \frac{z_2 - z_1}{z_1 + z_2}$ and $z_b = \frac{z_2 - x_1}{z_2}$, with $z_1 = 1$ and z_2 varying from 1 to 10 (see Equations (1) and (2) in the text). Green line: relationship between z_m and H_{MP}. Orange line: relationship between z_b and H_{BP}. Dotted line: H_{MP} or $H_{BP} = 0$. The dark green points show that a given H_{MP} value ($\approx$ 0.4) can be observed for very different H_{PR} values, and the same is true for H_{BP}.

Figure S2

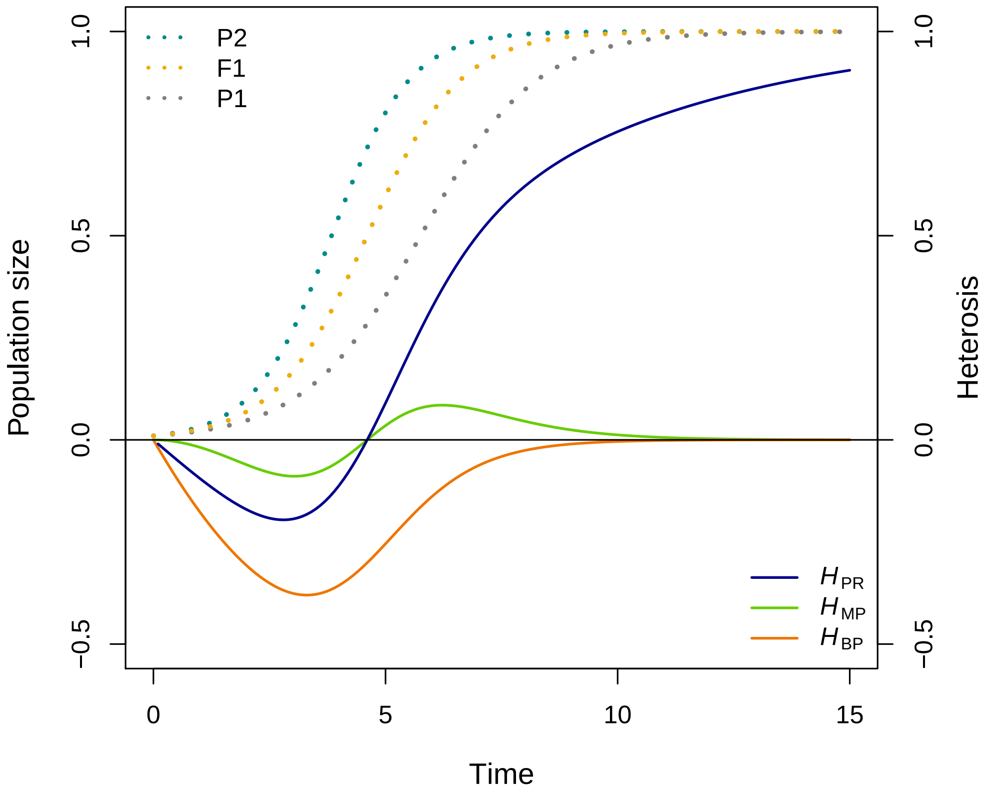

Heterosis for population size (simulations). Population sizes (dotted curves) follow over time a logistic function with $K = 1$ and $a = 100$ (see text). Parents P1 and P2 and hybrid F1 have respectively growth rates $r = 0.8$, $r = 1.2$ and $r = 1$ (i.e., this parameter is considered to be additive). Solid curves: profiles of heterosis coefficients (right scale).

© 2020 by the authors. Licensee MDPI, Basel, Switzerland. This article is an open access article distributed under the terms and conditions of the Creative Commons Attribution (CC BY) license (http://creativecommons.org/licenses/by/4.0/).
