# Supplementary material for: The Pitfalls of Heterosis Coefficients
Source: Plants (Basel). 2020 Jul 9;9(7):875. doi: 10.3390/plants9070875 (PMC7412094; doi:10.3390/plants9070875)
Supplement: Supplementary file 1 [file plants-09-00875-s001.zip › plants-835059_Suppl_Materials/Definitions/logo-mdpi-eps-converted-to.pdf]

MDPI
