# Supplementary material for: The Pitfalls of Heterosis Coefficients
Source: Plants (Basel). 2020 Jul 9;9(7):875. doi: 10.3390/plants9070875 (PMC7412094; doi:10.3390/plants9070875)
Supplement: Supplementary file 1 [file plants-09-00875-s001.zip › plants-835059_Suppl_Materials/Definitions/Table_Heterosis_Genetic_effects.pdf]

Reference	Coefficient	Coefficient as function of genetic effects
Mid-parent	$H_{\text{mp}} = z_{12} - \bar{z}$	$\sum d + \sum e_{\text{dom}} - \sum e_{\text{add even}}$
	$H_{\text{MP}} = \dfrac{z_{12} - \bar{z}}{\bar{z}}$	$\dfrac{\sum d + \sum e_{\text{dom}} - \sum e_{\text{add even}}}{\mu + \sum e_{\text{add even}}}$
	$H_{\text{PR}} = \dfrac{z_{12} - \bar{z}}{(z_2 - z_1)/2}$	$\dfrac{\sum d + \sum e_{\text{dom}} - \sum e_{\text{add even}}}{\sum a + \sum e_{\text{add odd}}}$
Best-parent	$H_{\text{bp}} = z_{12} - z_2$	$\sum d + \sum e_{\text{dom}} - \sum a - \sum e_{\text{add}}$
	$H_{\text{BP}} = \dfrac{z_{12} - z_2}{z_2}$	$\dfrac{\sum d + \sum e_{\text{dom}} - \sum a - \sum e_{\text{add}}}{\mu + \sum a + \sum e_{\text{add}}}$
