# Supplementary figures and images for: The Pitfalls of Heterosis Coefficients
Source: Plants (Basel). 2020 Jul 9;9(7):875. doi: 10.3390/plants9070875 (PMC7412094; doi:10.3390/plants9070875)

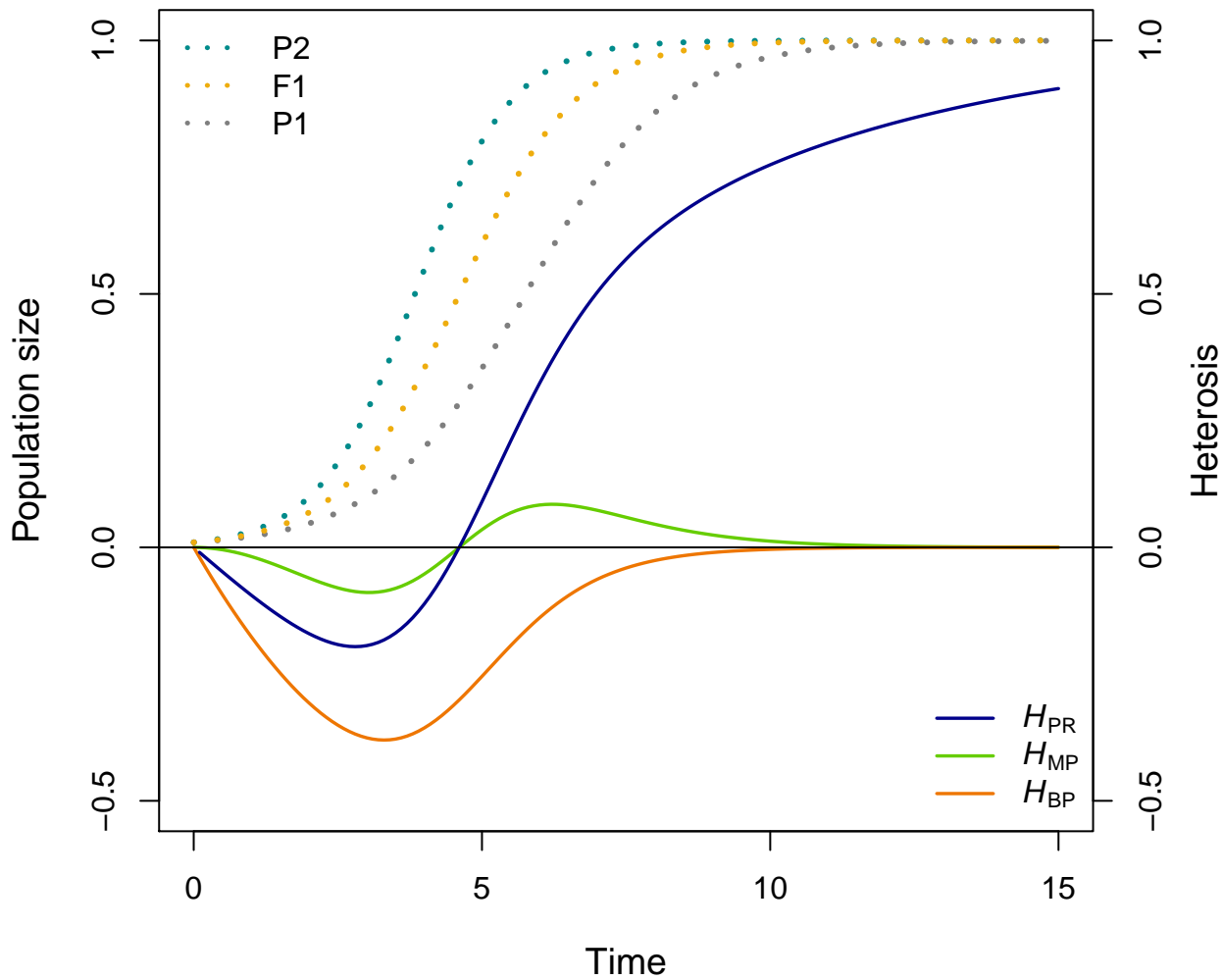

Supplement: Supplementary file 1 [file plants-09-00875-s001.zip › plants-835059_Suppl_Materials/Definitions/Logistic_Bis.pdf]

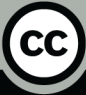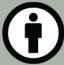

BY

Supplement: Supplementary file 1 [file plants-09-00875-s001.zip › plants-835059_Suppl_Materials/Definitions/logo-ccby-eps-converted-to.pdf]

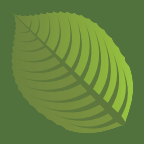

*plants*

Supplement: Supplementary file 1 [file plants-09-00875-s001.zip › plants-835059_Suppl_Materials/Definitions/plants-logo-eps-converted-to.pdf]

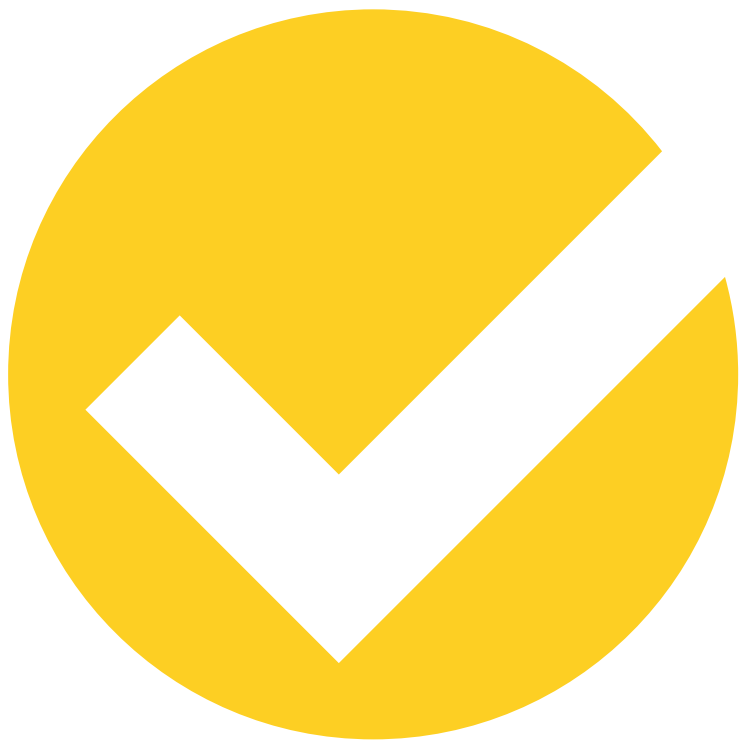

check for  
updates

Supplement: Supplementary file 1 [file plants-09-00875-s001.zip › plants-835059_Suppl_Materials/Definitions/logo-updates.pdf]

**A**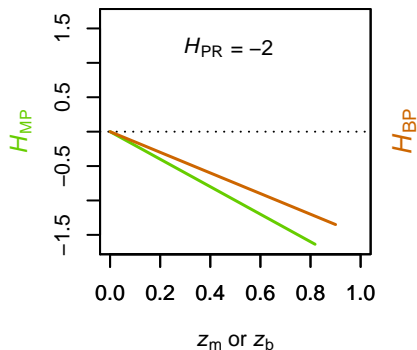**B**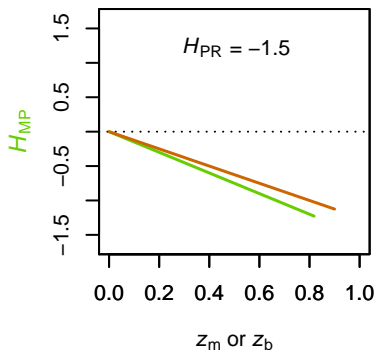**C**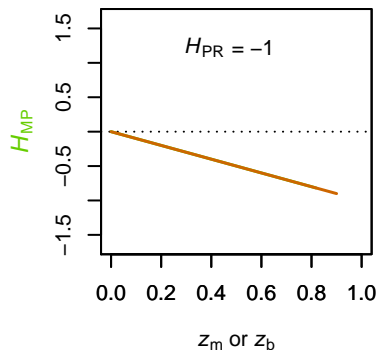**D**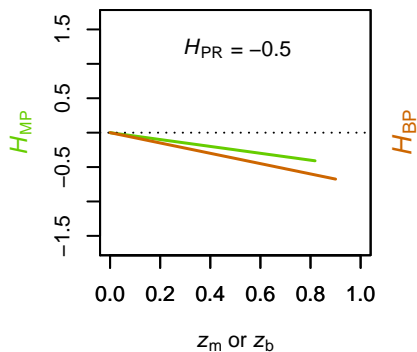**E**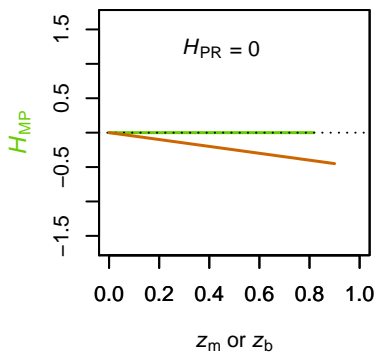**F**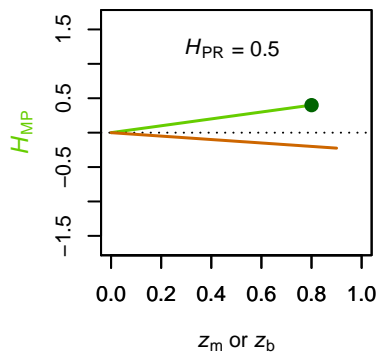**G**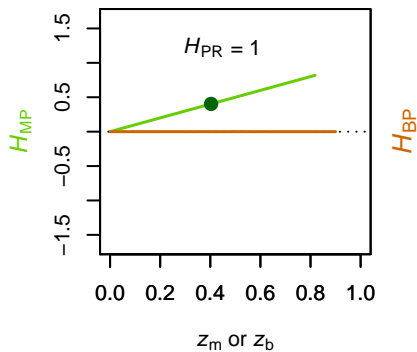**H**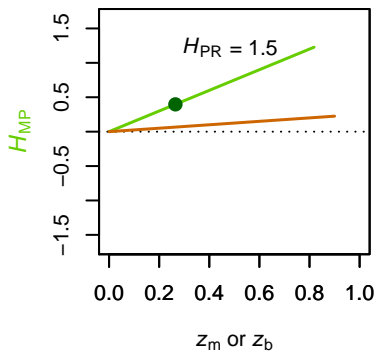**I**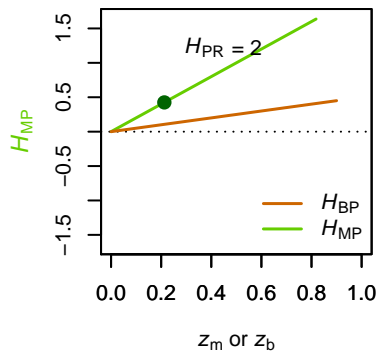

Supplement: Supplementary file 1 [file plants-09-00875-s001.zip › plants-835059_Suppl_Materials/Definitions/Relation_heterosis_cv-d_Bis.pdf]
